# Supplementary material for: Voluntary control of semantic neural representations by imagery with conflicting visual stimulation
Source: Commun Biol. 2022 Mar 18;5:214. doi: 10.1038/s42003-022-03137-x (PMC8933408; doi:10.1038/s42003-022-03137-x)
Supplement: Supplementary file 2 — Supplementary information [file 42003_2022_3137_MOESM2_ESM.pdf]

Supplementary Materials for

Voluntary control of semantic neural representations by imagery with conflicting visual stimulation

Ryohei Fukuma, Takufumi Yanagisawa,* Shinji Nishimoto, Hidenori Sugano, Kentaro Tamura, Shota Yamamoto, Yasushi Iimura, Yuya Fujita, Satoru Oshino, Naoki Tani, Naoko Koide–Majima, Yukiyasu Kamitani, Haruhiko Kishima

*Corresponding author: tyanagisawa@nsurg.med.osaka-u.ac.jp

This PDF file includes the following:

Supplementary Figs. 1 to 7
Supplementary Tables 1 to 4

Other Supplementary Materials for this manuscript include the following:

Supplementary Movies 1 to 4

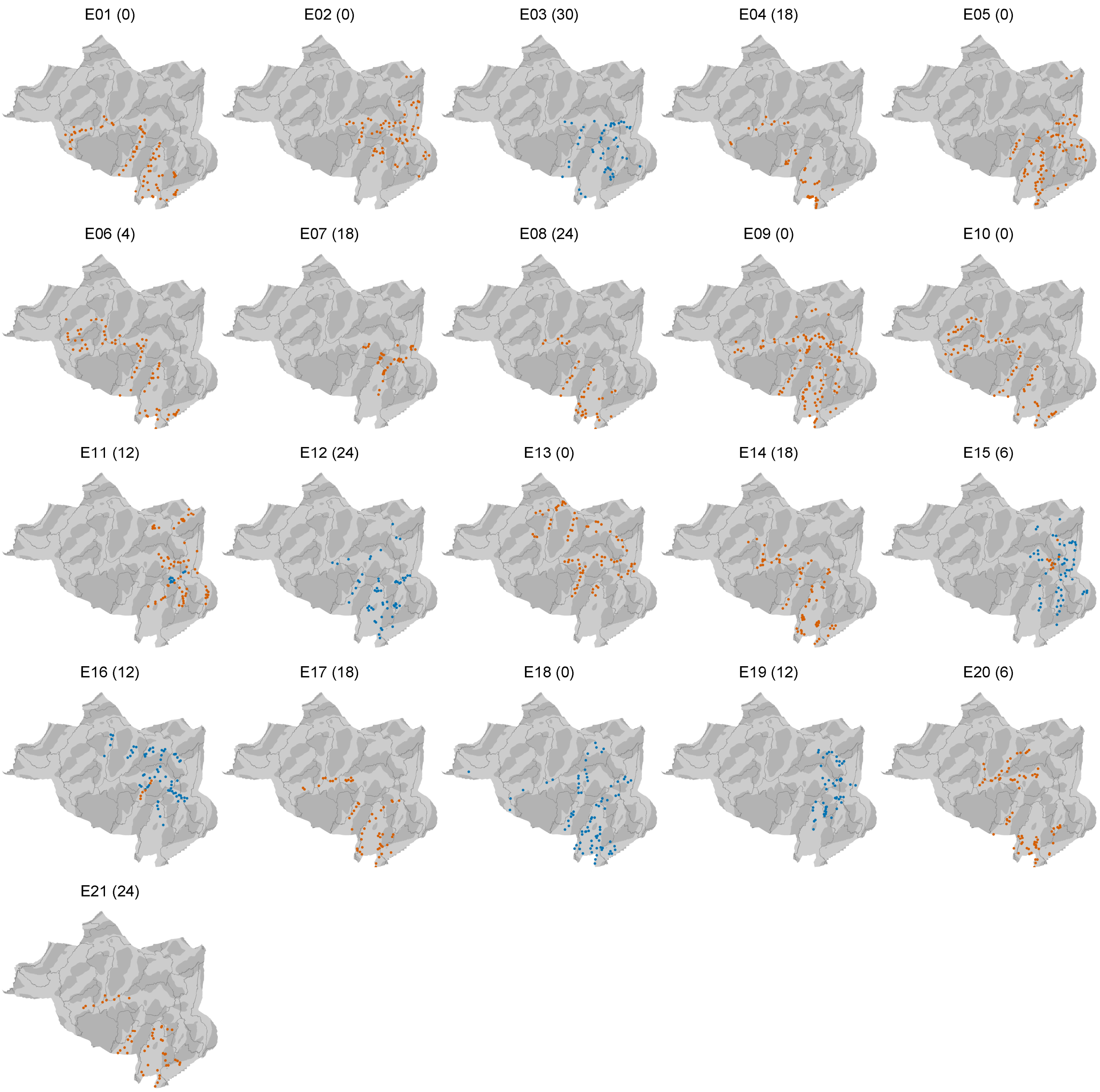

Supplementary Fig. 1. Individual locations of the electrodes. Locations of subdural electrodes for each subject are mapped on a normalized brain surface with red and blue color coding that denote electrodes in the left and right hemispheres, respectively. The number of depth electrodes is shown in parentheses.

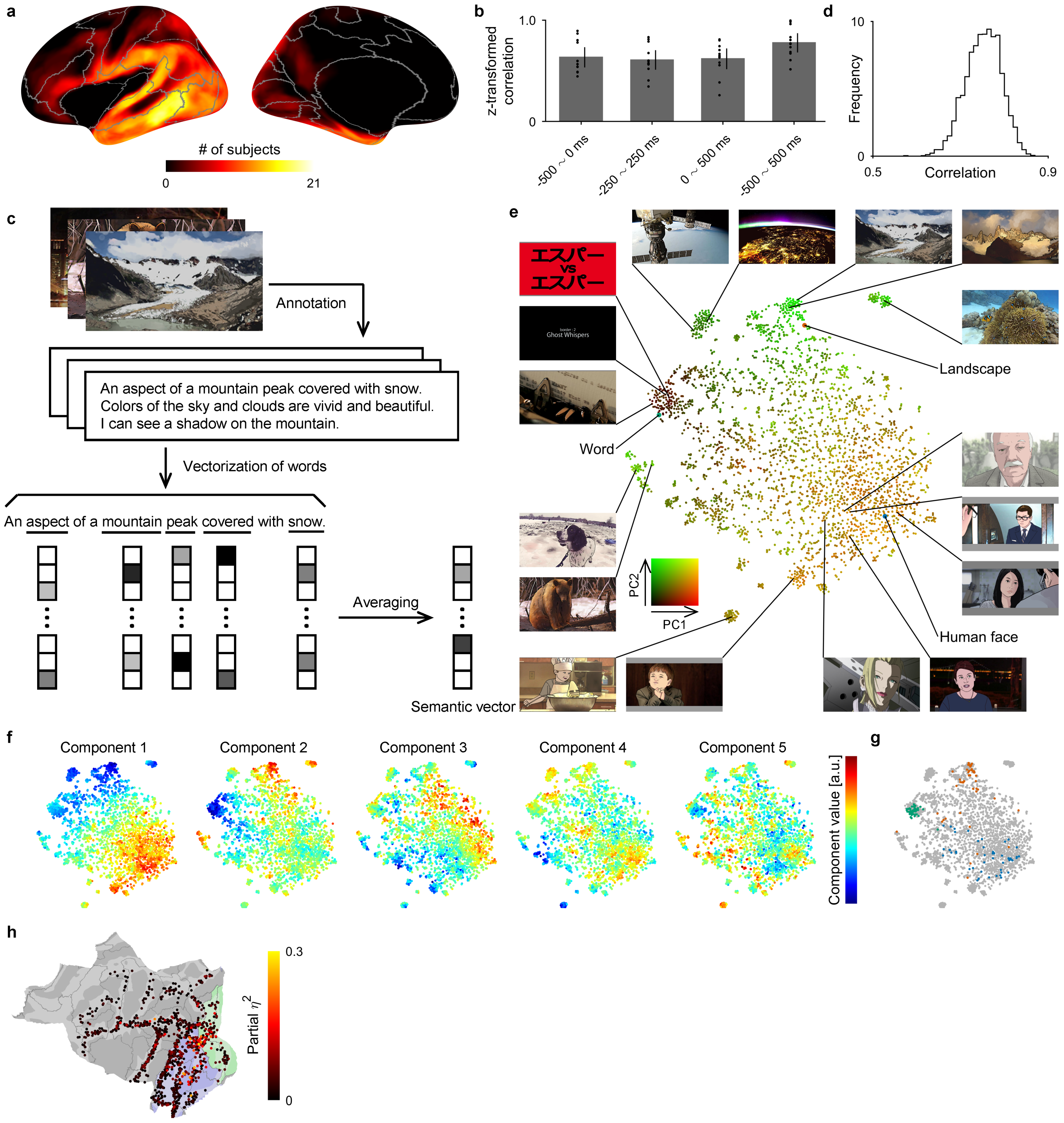

Supplementary Fig. 2. Location of electrodes, consistency of power in the high-γ band, and construction of semantic vectors. (a) Electrode coverage across subjects shown in Fig. 1a is color-coded on an inflated normalized brain to show the number of subjects with electrodes at each point. The electrode coverage map was created by averaging the maps of each subject. The locations of all electrodes in a subject were mapped to the left hemisphere of the normalized brain. By using the creepage distance on the pial surface of the template brain, an individual electrode coverage map was created. When a point on the surface of the left hemisphere was located within a distance of 10 mm from any subdural electrode, the coverage value of the point was set to 1; the coverage value of a point located further than 20 mm from all subdural electrodes was set to 0. For a point between these distances, a linearly

interpolated value was used according to the minimum distance to any of the subdural electrodes. (b) Subject-averaged consistency of powers in the high-γ band are shown as bars. To calculate the consistency, power in the high-γ band was calculated for all implanted electrodes using each of the time windows. The powers from all electrodes were then concatenated to calculate Pearson's correlation coefficients for all possible pairs across the four repetitions in the validation video; the correlation coefficients were Fisher z-transformed and averaged to acquire one value for a subject (shown as a black dot). Time 0 denotes the time when images from the validation video were annotated. Error bars denote 95% confidence intervals (CIs) among the subjects. (c) Each still image extracted from the videos was manually annotated and vectorized into semantic vectors using vector representations learned by the skip-gram model. (d) Consistency of semantic vectors across annotators is shown with a histogram. For each scene of the training videos, Pearson's correlation coefficients were calculated between all pairs of semantic vectors across five annotators (10 pairs) and were averaged within the scene to create the histogram. The mean of the distribution was 0.7523. (e) The dimensionality of the visual semantic space (1,000) was reduced to two dimensions for visualization using t-distributed stochastic neighbor embedding (t-SNE)[†] with the following parameters: number of initial dimensions by principal component analysis (PCA) pre-processing, 30; perplexity of the Gaussian kernel, 30. In addition to the 3,600 semantic vectors of the training videos, vector representations corresponding to "word", "landscape", and "human face" were included in the training dataset of the t-SNE. Each scene is shown as a point in the embedded space, with a color corresponding to its first and second principal components (PCs) of the semantic vectors of the training video. Illustrations are presented instead of the actual images used in the task. The green, red, and blue points denote points for vector representations of word, landscape, and human face, respectively. (f) The value of the first five PCs for each scene of the training videos is shown at the point of the embedding space in (e). (g) For the word, landscape, and human face categories, the 50 scenes that had the highest Pearson's correlation coefficient with their corresponding semantic vector (v_{word}, $v_{landscape}$, or v_{face}) are shown with green, red, and blue markers, respectively, in the embedding space (e). (h) Partial η^2 of Fig. 2c is color-coded and shown with dots at the location of each electrode.

† Maaten, L. v. d. & Hinton, G. Visualizing data using t-SNE. *Journal of machine learning research* **9**, 2579-2605 (2008).

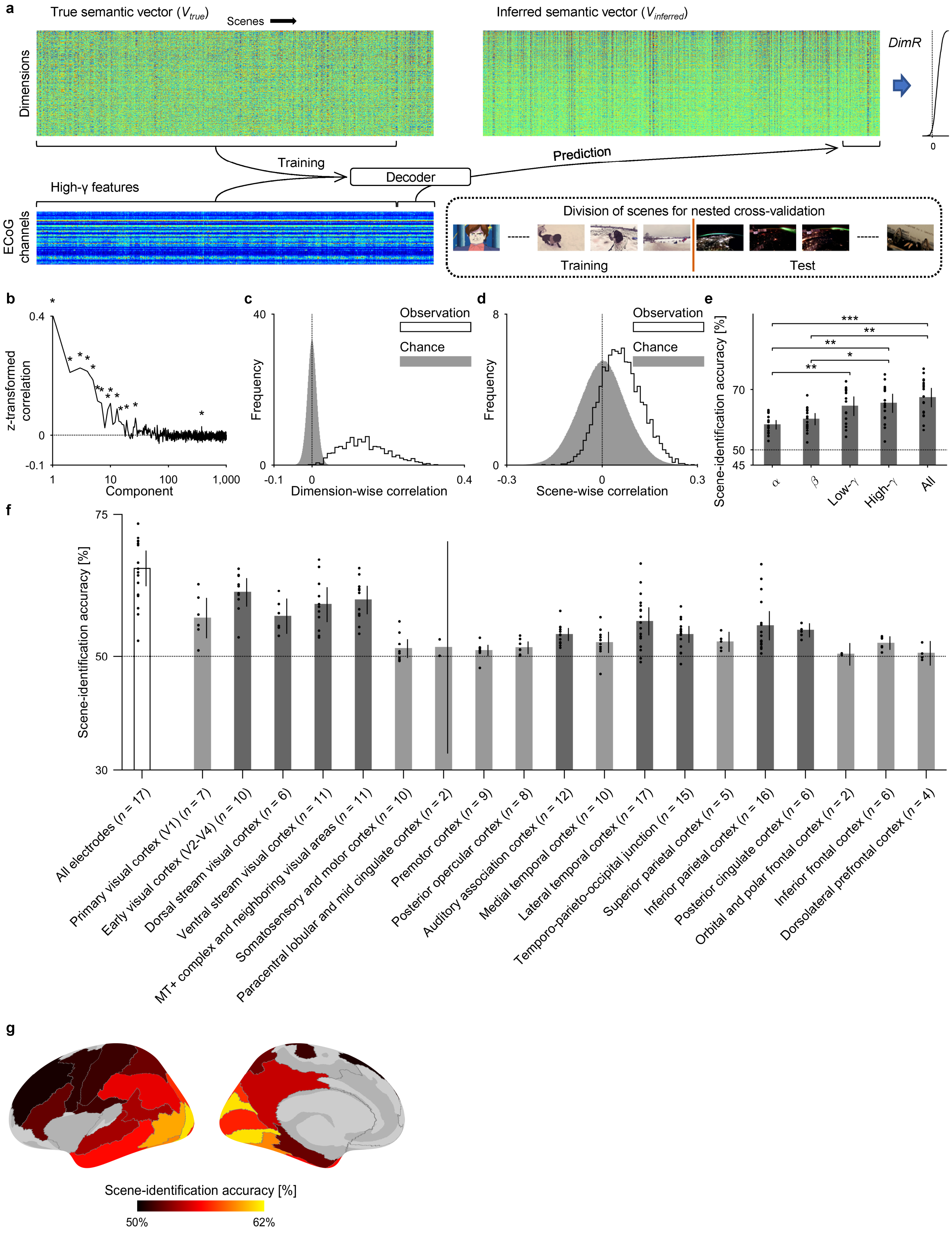
a
True semantic vector (V_{true})
Scenes
Dimensions
Inferred semantic vector ($V_{inferred}$)
DimR
0
Training
Decoder
Prediction
High-γ features
ECoG channels
Division of scenes for nested cross-validation
Training
Test
b
z-transformed correlation
Component
c
Observation
Chance
Frequency
Dimension-wise correlation
d
Observation
Chance
Frequency
Scene-wise correlation
e
Scene-identification accuracy [%]
α
β
Low-γ
High-γ
All
f
Scene-identification accuracy [%]
All electrodes (n = 17)
Primary visual cortex (V1) (n = 7)
Early visual cortex (V2-V4) (n = 10)
Dorsal stream visual cortex (n = 6)
Ventral stream visual cortex (n = 11)
MT+ complex and neighboring visual areas (n = 11)
Somatosensory and motor cortex (n = 10)
Paracentral lobular and mid cingulate cortex (n = 2)
Premotor cortex (n = 9)
Posterior opercular cortex (n = 8)
Auditory association cortex (n = 12)
Medial temporal cortex (n = 10)
Lateral temporal cortex (n = 17)
Temporo-parieto-occipital junction (n = 15)
Superior parietal cortex (n = 5)
Inferior parietal cortex (n = 16)
Posterior cingulate cortex (n = 6)
Orbital and polar frontal cortex (n = 2)
Inferior frontal cortex (n = 6)
Dorsolateral prefrontal cortex (n = 4)
g
Scene-identification accuracy [%]
50%
62%

Supplementary Fig. 3. Overview of decoding and decoding accuracy for the training videos. (a) An overview of the decoding of the semantic vectors using nested cross-validation is illustrated in the schematic. Entire scenes of the training videos were first divided into 10 groups without overlapping of the scenes between them; during the division, scenes from the same video source were kept in the same dataset so that there were no scenes from the same video source that belonged to multiple groups. Each of these 10 groups was used as a test dataset for decoding, and the other nine groups were used as a training dataset. For each test dataset, a decoder was trained with the high-γ features (left bottom column; representative example of E01) and the true semantic vectors of the training dataset (left upper column) to decode the high-γ features of the test dataset. *DimR*: dimension-wise correlation coefficients (see Methods). (b) For each principal component of the true semantic vectors of the training videos, the projected correlation coefficient ($\overline{z(PrjR^k(V_{inferred}, V_{true}))}$) is shown in order of the components. For visibility, the horizontal axis is shown in log space. Asterisks denote principal components that correlated significantly better than chance ($P < 0.5\times10^{-4}$ [Bonferroni-adjusted α-level; 0.05/1,000], $n = 17$, two-sided permutation test). (c) For each dimension of the visual semantic space (1,000), Pearson's correlation coefficients were calculated between the true semantic vectors and the inferred semantic vectors using high-γ features during the training videos to be Fisher z-transformed and averaged across all subjects ($n = 17$) (white). Chance distribution was estimated by shuffling scenes among the true semantic vectors in the same way that the projected correlation coefficients were tested (gray). (d) For each scene of the training videos, the scene-wise correlation coefficient of each subject was Fisher z-transformed and averaged ($n = 17$) (white). Chance distribution was estimated by shuffling scenes among the true semantic vectors in the same way that the projected correlation coefficients were tested (gray). (e) The subject-averaged scene-identification accuracies for all 3,600 scenes of the training videos are shown with a bar graph for four frequency bands (α: 8–13 Hz; β: 13–30 Hz; low γ: 30–80 Hz; high γ: 80–150 Hz) and their combinations. For decoding, features from all implanted electrodes were used. The scene-identification accuracy was calculated by comparing the inferred vector of each scene against the true semantic vectors of other scenes in the same test dataset in the nested cross-validation, but scenes originating from the same video source were removed; the scene-identification accuracies for all scenes were then averaged for each subject. Individual values are shown with dots. Error bars denote 95% CIs across subjects. *$P < 0.05$, **$P < 0.01$, and ***$P < 0.001$ ($P < 0.001$, $n = 17$ for each group, $F(4,80) = 8.55$, partial $\eta^2 = 0.299$, one-way analysis of variance [ANOVA] with the post hoc Tukey–Kramer test). (f) Subject-averaged scene-identification accuracies for all 3,600 scenes of the training videos using high-γ features are shown with a bar graph for each cortical region. Individual values are shown with dots. Error bars denote 95% CIs across subjects. Dark and light bars denote significant and non-significant, respectively, accuracies of the corresponding regions compared to chance (50%) ($P < 2.6\times10^{-3}$ [Bonferroni-adjusted α-level; 0.05/19], two-sided one-sample *t*-test). Scene-identification accuracies using high-γ features significantly differed depending on the cortical region ($P < 0.001$, $F(18,148) = 8.21$, partial $\eta^2 = 0.500$, one-way ANOVA with post hoc Tukey–Kramer tests). The scene-identification accuracies were significantly higher for the extrastriate early visual cortex (V2–V4), ventral stream visual cortex, and middle temporal complex and neighboring visual area than for the auditory association cortex ($P < 0.05$) and temporo-parieto-occipital junction ($P < 0.01$). Moreover, the accuracy with the V2–V4 was significantly higher than those with the lateral temporal, inferior parietal, and posterior cingulate cortices ($P < 0.05$). All of the aforementioned regions and the dorsal stream visual cortex showed significant scene-identification accuracies. MT+ complex: middle temporal complex. (g) The scene-identification accuracies averaged across subjects for each region (shown in (f)) were color-coded and plotted on the surface of the inflated

normalized brain, except the regions on which the electrodes were placed in fewer than three subjects.

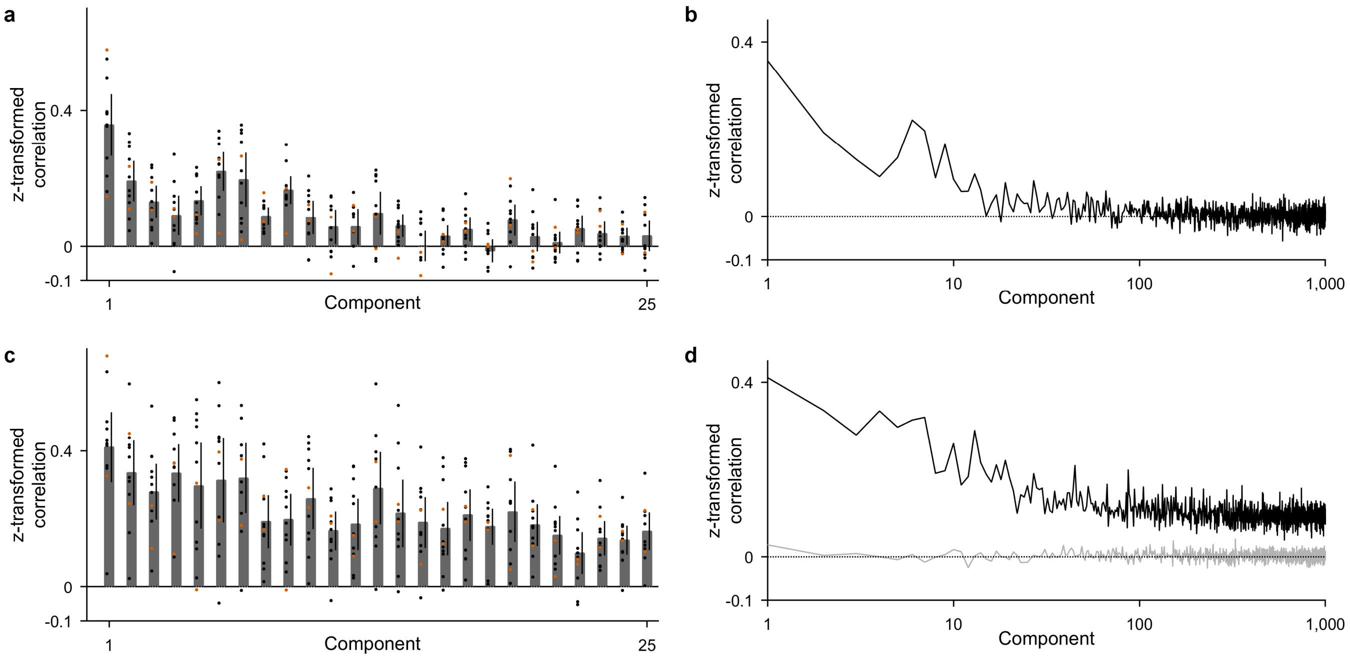

Supplementary Fig. 4. Decoding accuracy and consistency for the validation video. (a, b) From ECoGs obtained while watching the validation video, the high-γ features of each scene were acquired to infer the semantic vectors; then the inferred semantic vectors were evaluated for accuracy and consistency. For E01, the online decoder in the real-time feedback task was used to decode the high-γ features. The online decoder of E03 was re-trained excluding four electrodes that became noisy at the time of validation video presentation. For other subjects, decoders were trained using the high-γ features of all implanted electrodes for all 3,600 scenes of training videos by following the same procedures as the online decoders of E01 and E03. The projected correlation coefficients were evaluated by comparing the inferred vectors against the true semantic vectors of each scene created in exactly the same way as those for the training videos. For the projection, the direction vector of the PCA of the true semantic vectors of the training videos was used. The projected correlation coefficients were Fisher z-transformed and averaged across the subjects to be shown in (a) for the first 25 components and in (b) for all components. In (a), individual values of E01 and E03 and of those of other subjects are shown with red and black dots, respectively. Error bars denote 95% CIs among the subjects. In (b), the horizontal axis is shown in log space for visibility. Consistent with the crossover evaluation for the training video, the projected correlation coefficients were especially high for the first several principal components. (c, d) The consistency of the inferred semantic vector for the validation video was tested using replicability across the four repetitions in the validation video. The inferred semantic vectors were projected to the direction vector of the PCA determined from the semantic vectors of the training video to calculate the correlation coefficients for all possible pairs across the repetitions. The correlation coefficients were Fisher z-transformed and averaged to be shown in (c) for the first 25 components and in (d) for all components. In (c), individual values of E01 and E03 and of those of other subjects are shown with red and black dots, respectively. Error bars denote 95% CIs among the subjects. In (d), the horizontal axis is shown in log space for visibility. The gray line indicates chance-level consistency calculated by randomly shuffling the order of the inferred vectors within each repetition of the movies in the validation video 2,000 times. For the first several principal components, the Fisher z-transformed correlation

coefficients between the semantic vectors inferred from the high-γ features across the repeated 2.5-min videos were distributed at approximately 0.3–0.4.

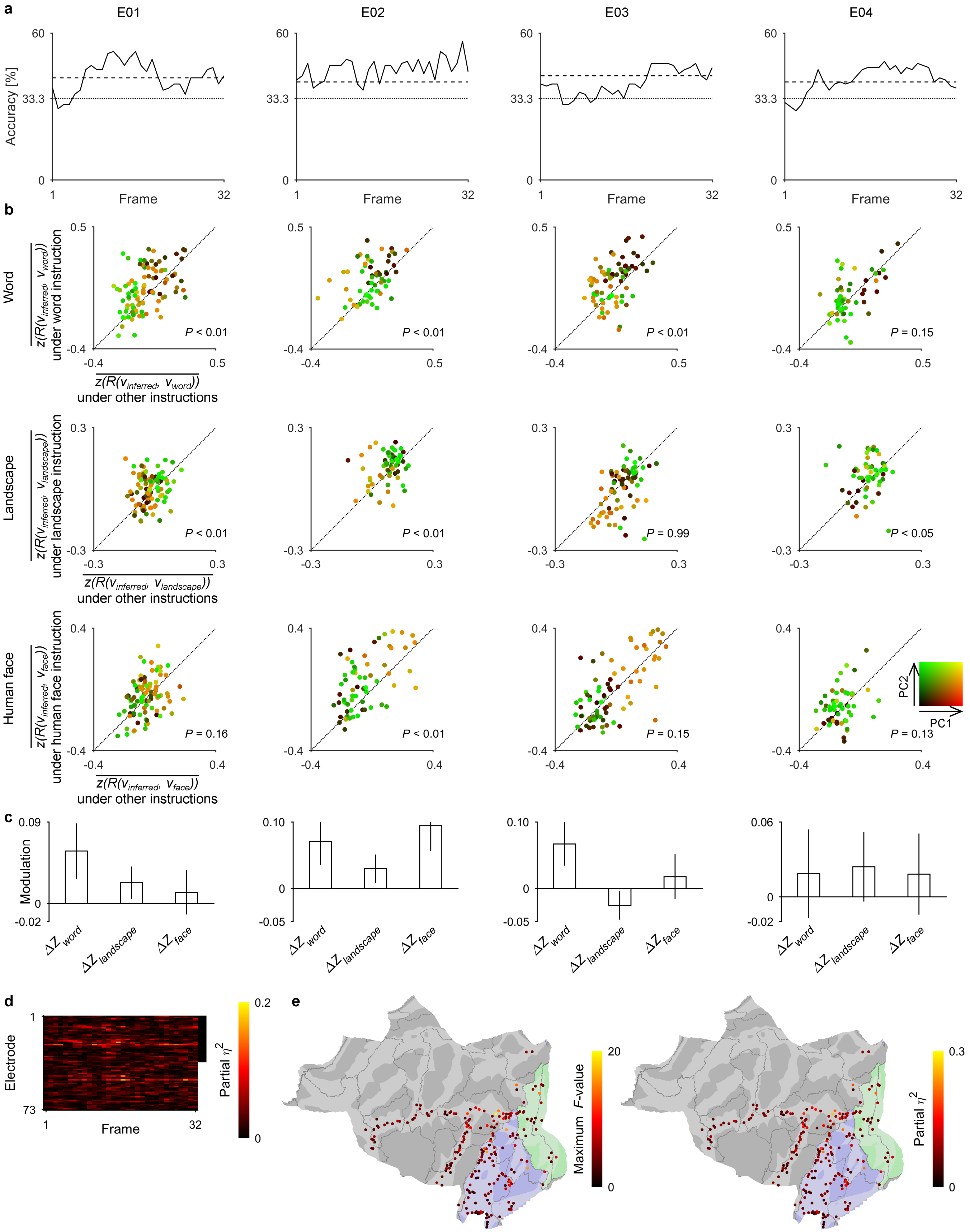

Supplementary Fig. 5. Accuracy and modulation in the real-time feedback task. (a) Each plot shows the time course of real-time decoding accuracy. For each frame in the real-time feedback task, an online vector (v_{online}) used to determine the feedback image was evaluated

as a three-choice task (three-choice accuracy). Dotted and dashed lines denote chance level (33.3%) and $P = 0.05$ false discovery rate-corrected significance levels, respectively. For the correction, the chance-level distribution of the three-choice accuracy with each frame was estimated by shuffling instructions of all trials 1 million times. (b, c) To elucidate how subjects controlled the inferred semantic vector during the real-time feedback task, modulation of the inferred vector depending on the instructed category (target category) was evaluated. During the real-time feedback task, the subjects viewed various images while visually imagining images representing the target category. For example, E01 viewed 124 different images commonly presented among three instructions; among them, 94 different images were included in the following analysis, avoiding overlaps of ECoGs corresponding to the images by rejection of a portion of the frames. Frames to be rejected were selected using a generic algorithm with a criterion of maximizing the number of images included in this analysis. For each frame, ECoGs from 0 to 1 s after the presentation of the image was decoded ($v_{inferred}$) using high-γ features to calculate scene-wise correlation coefficients with each semantic vector of the instructions (direction: word, landscape, or human face) ($R(v_{inferred}, v_{word})$, $R(v_{inferred}, v_{landscape})$ and $R(v_{inferred}, v_{face})$), which then were Fisher z-transformed and frame-averaged among the same image with the same instructions (instruction) ($Z_{direction}^{instruction} := \overline{z(R(v_{inferred}, v_{direction}))}$). In (b), the $Z_{direction}^{direction}$ for each image is shown on the vertical axis, where the average of two $Z_{direction}^{instruction}$ (instruction ≠ direction) is shown on the horizontal axis by a marker coded with color corresponding to its first and second principal components (PCs) of the semantic vectors of the training video. The target categories are shown on the left. In (c), the modulation of the inferred semantic vector towards one of the instructions for each image (e.g., ΔZ_{word} for "word" instruction) is calculated as the differences of $Z_{direction}^{instruction}$ among instructions (e.g., $\Delta Z_{word} := Z_{word}^{word} - (Z_{word}^{landscape} + Z_{word}^{face}) / 2$; that is, the value on the vertial axis in (b) subtracted with that on the horizontal axis). The ΔZ_{word} or $\Delta Z_{landscape}$ showed large modulation, suggesting the subjects succeeded in increasing the $R(v_{inferred}, v_{word})$ or $R(v_{inferred}, v_{landscape})$ with the corresponding instruction during the real-time feedback task. (d) Partial η^2 for Fig. 5e. (e) For four subjects (E01–E04), the maximum F-values of the high-γ powers in Fig. 5e and their corresponding partial η^2 were color-coded at the location of each electrode. High F-values were observed around the visual areas, suggesting that at least some portion of the electrodes with high F-values contributed to the control of the online vectors, making them closer to the semantic vector of the target category through changes in the high-γ power in this closed-loop condition.

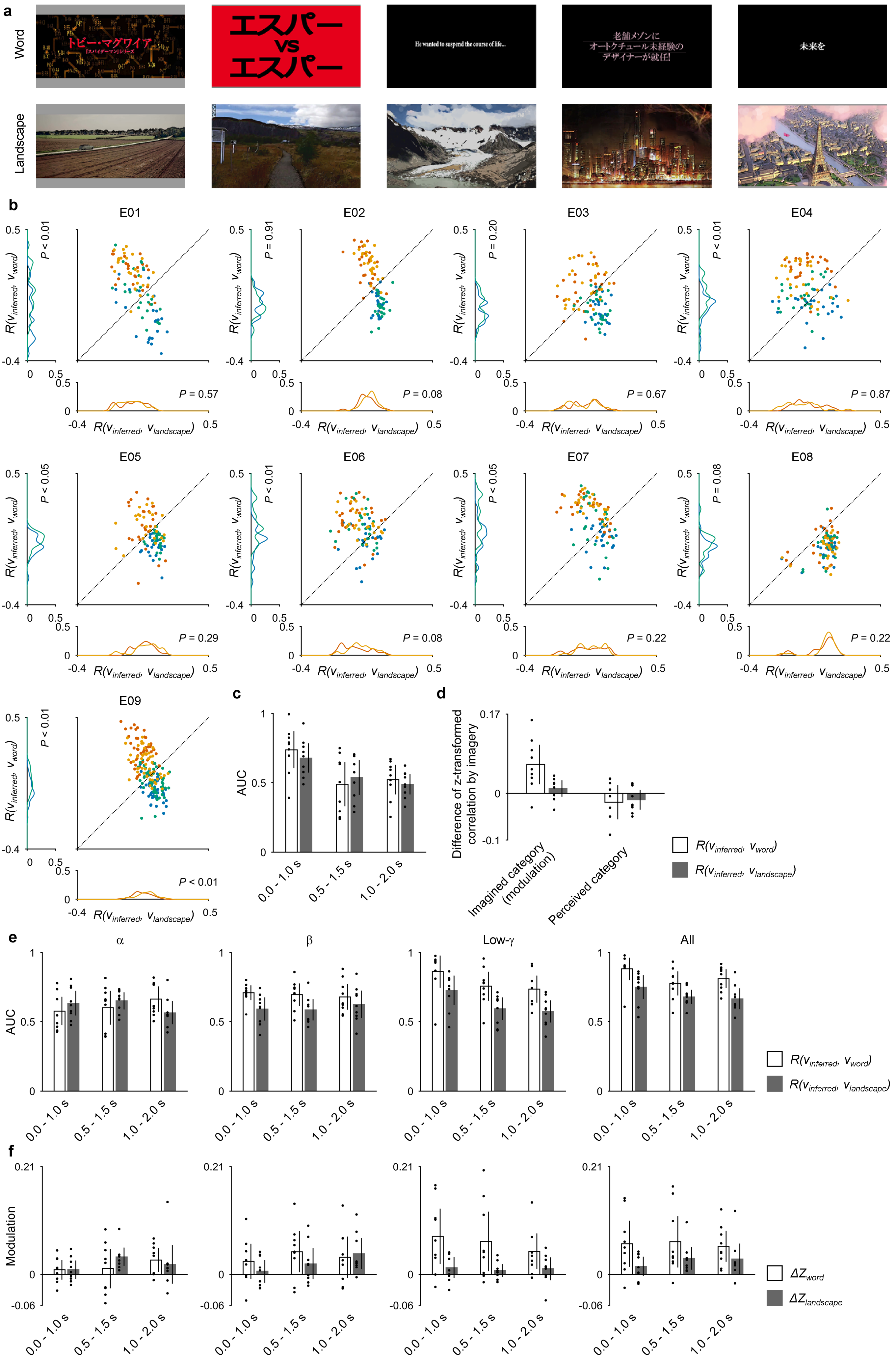
a
Word
Landscape
トビー・マグワイア
エスパー vs エスパー
He wanted to suspend the course of life...
老舗メゾンに
オートクチュール未経験の
デザイナーが就任!
未来を
b
E01 E02 E03 E04 E05 E06 E07 E08 E09
R(vinferred, vword)
R(vinferred, vlandscape)
P < 0.01
P = 0.91
P = 0.20
P = 0.57
P = 0.08
P = 0.67
P = 0.87
P < 0.05
P = 0.29
P = 0.22
P < 0.01
c
AUC
0.0 - 1.0 s
0.5 - 1.5 s
1.0 - 2.0 s
d
Difference of z-transformed correlation by imagery
Imagined category (modulation)
Perceived category
e
α
β
Low-γ
All
AUC
f
Modulation
ΔZword
ΔZlandscape

Supplementary Fig. 6. Image set for the imagery task and modulation of the inferred vector during the imagery task. (a) All images used in the imagery task are shown. Illustrations are presented instead of the actual images used in the task. (b) At the position of the corresponding Pearson's correlation coefficients with the semantic vector (v_{word} or $v_{landscape}$), markers representing one single presentation of an image (0 to 1 s) were plotted with color coding (red, presentation of word image; orange, presentation of word image while visually imagining landscape; blue, presentation of landscape image; green, presentation of landscape image while visually imagining word). The histogram in the subplot shows the estimated distribution of the correlation coefficients shown in the same color using the normal kernel (band width = 0.02). *P*-values were calculated using uncorrected one-sided Welch's *t*-test performed on the Fisher z-transformed correlation coefficients (n = 25 for each group, except E09 whose n = 50). We calculated the scene-identification accuracy by comparing the inferred vector of each presented image in a non-imagery period against the corresponding true semantic vector of the presented image and the vectors of other images in the same category. The accuracy for the word category was 54.11 ± 3.83% (mean ± 95% CIs among subjects); the accuracy for the landscape category was 50.28 ± 4.57%. Replicability of the inferred semantic vectors in the non-imagery period was evaluated by calculating the Pearson's correlation coefficients for all possible pairs among presentations of the same image. The average of Fisher z-transformed correlation coefficients was 0.8619 ± 0.3308 (mean ± 95% CIs among subjects) for the landscape images and 0.6443 ± 0.1722 for the word images. (c) For each 1-s time window in the imagery period, the AUC to identify the category of the presented image (word or landscape) is shown solely using $R(v_{inferred}, v_{word})$ (white bars) or $R(v_{inferred}, v_{landscape})$ (black bars). Individual values are shown with dots. Error bars denote the 95% CIs among subjects (n = 9). (d) The difference in the correlation coefficients in (b) attributable to the imagery (0–1 s of the imagery period – 0–1 s of the non-imagery period) was evaluated for the semantic vector (v_{word} or $v_{landscape}$) of the imagined or perceived categories, and was averaged among the subjects. The difference for the imagined category is exactly same as the modulation in Fig. 7c. Individual values are shown with dots. Error bars denote the 95% CIs among subjects (n = 9). For both the perceived word and landscape images, the difference was not significantly different from zero (word: P = 0.2636, $t(8)$ = -1.20, n = 9, uncorrected two-sided one-sample *t*-test; landscape: P = 0.1777, $t(8)$ = -1.48). (e, f) For each 1-s time window, ECoGs were decoded using features from three frequency bands (α: 8–13 Hz; β: 13–30 Hz; low γ: 30–80 Hz) and combinations of them with the high-γ band to show (e) the area under the curve (AUC) to identify the category of the presented image in the non-imagery period (word or landscape) and (f) modulation. Individual values are shown with dots. Error bars denote 95% CIs among subjects (n = 9).

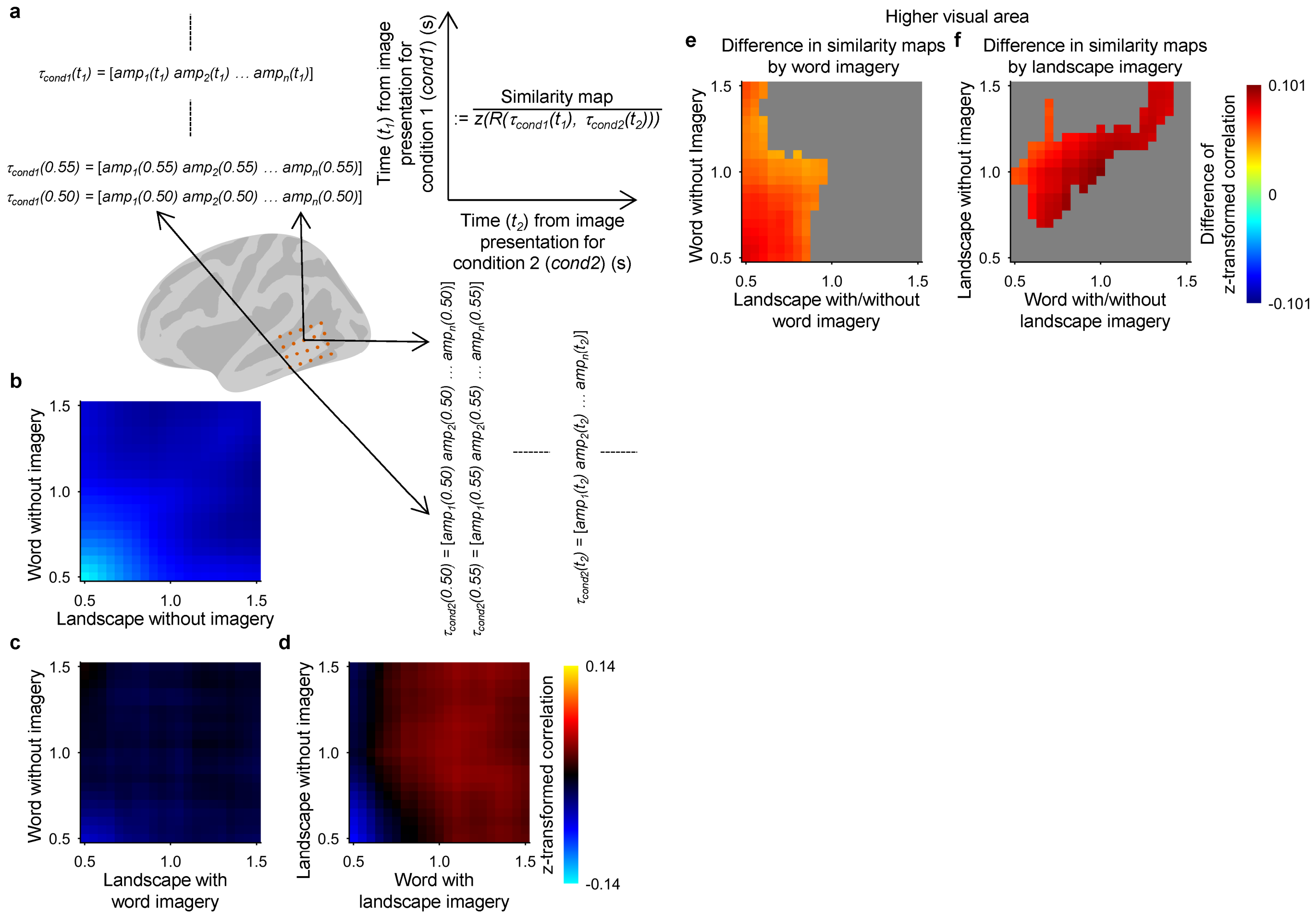

Supplementary Fig. 7. Similarity maps for high-γ amplitudes during the imagery task. (a) The similarity in neural activity during the non-imagery and imagery periods was evaluated using amplitude in the high-γ band for all subjects who participated in the imagery task (E01–E09 and E18–E21). For each channel of subdural electrodes, pre-processed ECoGs were converted to instantaneous amplitudes by band-pass filtering, Hilbert transformation, and taking its absolute value. For band-pass filtering, a finite impulse response (FIR) filter was applied by the pop_eegfiltnew function in EEGLAB software[†]. The order of the filter was 1,651, which was determined automatically by the function. Hilbert transformation was also applied by an FIR filter with the order of 413 (empirically determined; one-fourth of the order of the band-pass filter). Although each image was presented for 2,000 ms, to avoid inference from previous or next presentation of the image induced by the FIR filtering, only instantaneous amplitudes from 250 to 1,750 ms after image presentation were included in the analysis. To remove baseline activity due to the presentation of visual stimuli, the average of the instantaneous amplitudes during the image presentation in the non-imagery period was subtracted from the instantaneous amplitude of each image presentation. The instantaneous amplitude was then averaged within 500-ms time windows spaced every 50 ms (90% overlap) (denoted as $amp_k(t)$ for the high-γ amplitude of the k-th electrode (k=1,…n)). The amplitude vector at time t of the i-th trial in the task condition (*cond*, non-imagery/imagery period, and stimulus image of word/landscape) was defined as $\tau_{cond}(i, t)$. Pearson's correlation coefficient was calculated among amplitude vectors ($\tau_{cond1}(i, t_1)$ and $\tau_{cond2}(j, t_2)$) from paired trials in two task conditions (*cond1* and *cond2*) and at each pair of time points in the two conditions (t_1 for *cond1* and t_2 for *cond2*); then the similarity map was quantified by averaging Fisher z-transformed Pearson's correlation coefficients for pairs of trials ($\{(i,j) \in Pair(cond1, cond2)\}$):

$$\frac{1}{|Pair(cond1, cond2)|}\sum_{(i,j)\in Pair(cond1,cond2)} z(R(\tau_{cond1}(i,t_1), \tau_{cond2}(j,t_2))).$$

All possible pairs of trials were included in the analysis for the similarity map between the non-imagery period with the word image and that with the landscape image (625 pairs except E09, whose number of pairs was 2,500). For the similarity map between the non-imagery period and imagery period, each trial in the imagery period was paired with the trials in the non-imagery period in which the subject watched the same image as the imagined one (125 pairs except E09, whose number of pairs was 500). Finally, the similarity maps of each subject were averaged among the subjects, resulting in a similarity map of the neural representations. (b–d) Similarity map between different pairs of conditions for higher visual area ($n = 13$). The paired conditions are (b) watching the word and landscape image without imagery, (c) watching the word image without imagery and the landscape image with word imagery, and (d) watching the landscape image without imagery and the word image with landscape imagery. Time 0 on each axis corresponds to the presentation of the image. (e, f) Significant differences in two similarity maps are shown with color coding for higher visual area ($n = 13$) ($P < 0.01$, uncorrected two-sided one-sample t-test). Map (e) shows map (c) – map (b), and map (f) shows map (d) – transverse of map (b). The same analysis performed for the early visual area ($n = 7$) resulted in no significant differences.

† Delorme, A. & Makeig, S. EEGLAB: An open source toolbox for analysis of single-trial EEG dynamics including independent component analysis. *J Neurosci Methods* **134**, 9-21 (2004).

Supplementary Table 1. Number of electrodes for each cortical region.

	E01	E02	E03	E04	E05	E06	E07	E08	E09	E10	E11	E12	E13	E14	E15	E16	E17	E18	E19	E20	E21
Primary visual cortex (V1)	0	6	0	0	5	0	1	0	2	0	8	0	2	0	8	0	0	0	1	0	0
Early visual cortex (V2–V4)	0	14	5	0	16	0	6	0	4	0	12	5	7	0	15	2	0	4	7	0	0
Dorsal stream visual cortex	0	3	0	0	1	0	0	0	0	0	0	1	4	0	7	4	0	0	6	0	0
Ventral stream visual cortex	0	4	6	0	3	0	2	1	4	0	11	11	0	0	10	1	1	0	3	4	0
MT+ complex and neighboring visual areas	0	5	4	0	7	0	9	0	6	0	14	0	2	4	5	7	2	5	13	0	2
Somatosensory and motor cortex	2	0	0	2	0	3	0	3	2	2	0	0	14	2	0	9	1	1	0	11	1
Paracentral lobular and mid cingulate cortex	0	0	0	0	0	0	0	0	0	0	0	0	8	0	0	3	0	0	0	0	0
Premotor cortex	3	0	0	1	0	6	0	0	1	4	0	0	4	5	0	6	3	0	0	2	2
Posterior opercular cortex	1	0	0	2	0	1	0	1	4	1	0	2	0	6	0	0	2	1	0	3	2
Auditory association cortex	10	0	5	6	4	5	0	8	8	8	0	6	5	6	0	0	8	10	0	8	9
Medial temporal cortex	7	0	0	1	5	7	0	1	2	5	0	1	0	5	1	0	4	5	0	4	5
Lateral temporal cortex	29	9	13	23	31	18	7	22	33	17	5	20	5	27	4	4	24	33	6	28	23
Temporo-parieto-occipital junction	5	4	4	0	7	3	6	1	3	3	0	4	5	4	2	5	1	5	2	1	0
Superior parietal cortex	0	0	0	0	0	0	0	0	3	0	5	0	6	0	2	5	0	6	6	3	0
Inferior parietal cortex	4	17	13	2	1	7	5	3	27	6	10	4	16	6	12	25	6	8	5	14	1
Posterior cingulate cortex	0	2	0	0	2	0	0	0	0	0	11	2	0	0	2	1	0	0	1	0	0
Orbital and polar frontal cortex	4	0	0	0	0	0	0	0	0	2	0	0	0	0	0	0	0	2	0	0	0
Inferior frontal cortex	9	0	0	3	0	7	0	0	5	7	0	0	0	0	0	0	2	2	0	0	3
Dorsolateral prefrontal cortex	0	0	0	0	0	9	0	0	0	11	0	0	2	1	0	0	0	0	0	0	0
Depth	0	0	30	18	0	4	18	24	0	0	12	24	0	18	6	12	18	0	12	6	24

MT+ complex: middle temporal complex.

Supplementary Table 2. Words that have the highest and lowest correlation coefficients with principal components

Order of component	Top 10 words	Bottom 10 words
1st	Male, Female, Wearing, Hairstyle, Blond hair, Older, Clothing, Wearing, Appearance, Girls	Terrain, Slope, Terrace, East side, North side, Gradient, Nearby, Hills, Landscape, Rainfall
2nd	Climb, Walking, Foot, Lake, Sea, Mountaintop, Snow, Mountain, Cliff, Slope	Text[1], Text[2], Write, Display, Screen, Explicit, Telop, Font, Input, Logo
3rd	Same building, Construction, Accommodation, Neighborhood, Relocation, City, Resident, Immigration, Building, Government	White, Black, Red, Yellow[3], Light blue, Color, Green, Blue, Hair, Yellow[4]
4th	Look, Feel, Expression, Feeling, Capturing, Dark, Gentle, Looking, Impression, Understanding	Furniture, Confectionery, Supplies, Shop, Cooking, Selling, Table, Restaurant, Dish, Box lunch
5th	*No*[5], What, Say, Self, to be able to do, to do, to eat, to eat, to be troubled	Keys, Color[2], Logo, Middle, Men, Light blue, Gothic, Costume, Color[6], Uniform
6th	Front, Both sides, Rear, Back, Inner, Head, Mounting, Outer, Top, Back	Fun, Happy, Girls, Friends, Themes, I, Loving, Singing, Loving, Boys
7th	Window, Table, Room, Ceiling, Sitting, Bed, Stairs, Chair, Entrance, Door	Conflict, Force, Capture, Strength, Arrest, Weapon, Defeat, Exercise, Guerrilla, Led
8th	Writing[7], Mountain, Mountains, Foot, Tradition, Writing[7], Descending, Pass, Altitude, Tributary	Earth, Spacecraft, Space, Infrared, Room, Planet, Glowing, Solar system, Liquid crystal, Camera
9th	Male, Female, Fish[8], Fish[8], Ocean, Edible, Collection, Seawater, Food, Extraction	Cry, Go home, Boy, Walk, Come, Go, I, You, Ask, When
10th	Wearing[7], Clothes, Clothing, *No*[5], Uniform, Think[7], Think[7], Know, Reason, Wearing[7]	Character (in book, film, etc.), Crash, Animation, Draw, Mecha, Drawing, Fish, Shoot, Illustration, Robot

From the text corpus used to train the skip-gram model, the most frequent 10,000 words were selected as candidates; their vector representations were tested by Pearson's correlation coefficients with the first 10 principal components of the true semantic vectors. [1] In Kanji characters; [2] In Katakana characters; [3] Noun; [4] Adjectives; [5] Japanese particles; [6] Not translated because word was in learned English; [7] Translated from different Japanese verbs; [8] Translated from different Japanese nouns.

Supplementary Table 3. Comments after each session of the real-time feedback task

Subject	Session	Comments
E01	1st	Somehow, I could control. Small texts were displayed for the word instruction. I was surprised when an image similar to what I intended showed up. Human face is the easiest to show, followed by landscape. I could display word images well, too.
	2nd	Images changed in a moment, and I thought "oh". It was difficult to catch up with the change in the screen, I think I performed better in the 1st session.
	3rd	I could somehow control, but there are doubtful images. Word was the easiest, followed by human face. I imagined what I wanted to display, not a certain image. For the word instruction, I imagined an image with subtitles, where I just imagined human and landscape for the human face and the landscape instructions, respectively. When I focused on the displayed image, a similar image came out (human face and subtitles).
	4th	I don't know in which session I performed best. I could control if I focused on a similar element in the displayed image.
E02	1st	It is difficult to imagine a human face. Landscape is easy to display, where word is difficult. To display landscape, I imagined an image of a mountain. Other categories may be difficult because of the number of the different images in categories such as subtitles. I imagined a baby for human face, but it was difficult to display. It became easier to control than the first time.
	2nd	It was difficult to display either word or human face, which I forgot. I imagined a baby for human face instructions and subtitles for word.
	3rd	Word was the most difficult category to display. Images with word come up but only briefly. Human face became easier to show.
	4th	I could not get the knack, although I think I got better control after the first time. I could display landscape from the first time. Word was difficult. I got better with human face. I think I could display an image of the face of a computer-graphic girl better.
E03	1st	When I intended to display, the images wouldn't come; when I didn't intend, they came. It seemed that the images came out contrariwise.
	2nd	The images did not come out when I intended.
	3rd	This time, images with landscape and word displayed contrariwise. I performed better in the 2nd session. When I tried to display word, landscape was displayed in this session, where images with word had correctly displayed in the last session.
	4th	I performed while thinking that I could really display. Without thinking, I could display word. Human face and landscape were contrariwise. But, I imagined as usual. Landscape image came out when I imagined faces of many people, and so I tried to imagine the opposite way, but it did not work.
E04	1st	I could display landscape best, followed by word. Human face was difficult. For the landscape instruction, I imagined an image of mountains and sea.
	2nd	There was little difference from the 1st session.
	3rd	I could control a little better. I could display several images with mountains for the landscape instruction. I searched for the subtitles for the word instruction. For the human face, I imagined the baby wearing yellow cloth.
	4th	I think I succeeded to control. For the landscape instruction, images with mountains showed up. For the word, subtitles. I could display the images with a human face along with imagining subtitles.

Supplementary Table 4-1. Confusion matrix at the time point when classification accuracy peaked in the real-time feedback task for E01

		Predicted		
		Word	Landscape	Human face
Instruction	Word	26	6	8
	Landscape	10	24	6
	Human face	18	9	13

Supplementary Table 4-2. Confusion matrix at the time point when classification accuracy peaked in the real-time feedback task for E02

		Predicted		
		Word	Landscape	Human face
Instruction	Word	16	16	8
	Landscape	5	28	7
	Human face	2	14	24

Supplementary Table 4-3. Confusion matrix at the time point when classification accuracy peaked in the real-time feedback task for E03

		Predicted		
		Word	Landscape	Human face
Instruction	Word	25	10	5
	Landscape	10	17	13
	Human face	12	13	15

Supplementary Table 4-4. Confusion matrix at the time point when classification accuracy peaked in the real-time feedback task for E04

		Predicted		
		Word	Landscape	Human face
Instruction	Word	17	21	2
	Landscape	5	34	1
	Human face	9	24	7
