# Supplementary material for: Voluntary control of semantic neural representations by imagery with conflicting visual stimulation
Source: Commun Biol. 2022 Mar 18;5:214. doi: 10.1038/s42003-022-03137-x (PMC8933408; doi:10.1038/s42003-022-03137-x)
Supplement: Supplementary file 3 — Description of Additional Supplementary Files [file 42003_2022_3137_MOESM3_ESM.pdf]

Description of Additional Supplementary Files

File name: Supplementary Movie 1.
Description: Representative trials in a real-time feedback task. From the real-time feedback task with E01, representative trials, including the trials shown in Fig. 5a, were reconstructed from log files as a movie. Illustrations are presented instead of the actual images used in the task.

File name: Supplementary Movie 2.
Description: Visualization of online vectors in a real-time feedback task. For the representative trials in Supplementary Movie 1 (upper-right panel), the first and second principal components of the semantic vector for each feedback image are marked with a yellow dot (left panel). Scene-wise correlation coefficients between the online vector and the semantic vectors of the three categories are also shown (bottomright panel).

File name: Supplementary Movie 3.
Description: Video from the real-time feedback task with E01. For the representative trials in Supplementary Movie 1, video recorded during the real-time feedback task performed by E01 is shown. In the video, E01 is seated on the left side but was masked by the captions for instructions.

File name: Supplementary Movie 4.
Description: Video from the real-time feedback task with E02. The video recorded during the real-time feedback task performed by E02 is shown for representative trials. In the video, E02 is seated on the left side but was masked by the captions for instructions.
