# Supplementary material for: Voluntary control of semantic neural representations by imagery with conflicting visual stimulation
Source: Commun Biol. 2022 Mar 18;5:214. doi: 10.1038/s42003-022-03137-x (PMC8933408; doi:10.1038/s42003-022-03137-x)
Supplement: Supplementary file 8 — Reporting Summary [file 42003_2022_3137_MOESM8_ESM.pdf]

nature portfolio

Corresponding author(s): Takufumi Yanagisawa

Last updated by author(s): Feb 4, 2022

Reporting Summary

Nature Portfolio wishes to improve the reproducibility of the work that we publish. This form provides structure for consistency and transparency in reporting. For further information on Nature Portfolio policies, see our Editorial Policies and the Editorial Policy Checklist.

Statistics

For all statistical analyses, confirm that the following items are present in the figure legend, table legend, main text, or Methods section.

n/a	Confirmed	
☐	☒	The exact sample size (*n*) for each experimental group/condition, given as a discrete number and unit of measurement
☐	☒	A statement on whether measurements were taken from distinct samples or whether the same sample was measured repeatedly
☐	☒	The statistical test(s) used AND whether they are one- or two-sided<br>*Only common tests should be described solely by name; describe more complex techniques in the Methods section.*
☐	☒	A description of all covariates tested
☐	☒	A description of any assumptions or corrections, such as tests of normality and adjustment for multiple comparisons
☐	☒	A full description of the statistical parameters including central tendency (e.g. means) or other basic estimates (e.g. regression coefficient) AND variation (e.g. standard deviation) or associated estimates of uncertainty (e.g. confidence intervals)
☐	☒	For null hypothesis testing, the test statistic (e.g. *F*, *t*, *r*) with confidence intervals, effect sizes, degrees of freedom and *P* value noted<br>*Give P values as exact values whenever suitable.*
☒	☐	For Bayesian analysis, information on the choice of priors and Markov chain Monte Carlo settings
☒	☐	For hierarchical and complex designs, identification of the appropriate level for tests and full reporting of outcomes
☐	☒	Estimates of effect sizes (e.g. Cohen's *d*, Pearson's *r*), indicating how they were calculated

Our web collection on statistics for biologists contains articles on many of the points above.

Software and code

Policy information about availability of computer code

Data collection	No software was used.
Data analysis	MATLAB R2015b

For manuscripts utilizing custom algorithms or software that are central to the research but not yet described in published literature, software must be made available to editors and reviewers. We strongly encourage code deposition in a community repository (e.g. GitHub). See the Nature Portfolio guidelines for submitting code & software for further information.

Data

Policy information about availability of data

All manuscripts must include a data availability statement. This statement should provide the following information, where applicable:

- Accession codes, unique identifiers, or web links for publicly available datasets
- A description of any restrictions on data availability
- For clinical datasets or third party data, please ensure that the statement adheres to our policy

All data are available in the main text, the supplementary materials, or figshare (https://figshare.com/articles/dataset/Datasets_for_Fukuma_et_al_Communications_Biology/12916037).

Field-specific reporting

Please select the one below that is the best fit for your research. If you are not sure, read the appropriate sections before making your selection.

☒ Life sciences ☐ Behavioural & social sciences ☐ Ecological, evolutionary & environmental sciences

For a reference copy of the document with all sections, see nature.com/documents/nr-reporting-summary-flat.pdf

Life sciences study design

All studies must disclose on these points even when the disclosure is negative.

Sample size	The amount of data collected per patient depended on their clinical treatment schedule and the amount of time each participant was willing to volunteer for the study. According to our previous work, the duration of the training videos in the video-watching task and the number of trials in the real-time feedback and imagery tasks were determined to be sufficient to train a decoder and to show the modulation of the neural activities in their corresponding analyses, respectively.
Data exclusions	No data were excluded from analysis.
Replication	Reproducibility of the control of semantic vectors in the real-time feedback task was confirmed with four independent study participants. We confirmed replication of the results among the four participants.
Randomization	This study is a single arm, proof-of-concept study without randomized grouping of interventions. Therefore, we did not randomize the subjects in this study.
Blinding	Because there are no group allocations in this study, we did not perform the blinding in this study.

Reporting for specific materials, systems and methods

We require information from authors about some types of materials, experimental systems and methods used in many studies. Here, indicate whether each material, system or method listed is relevant to your study. If you are not sure if a list item applies to your research, read the appropriate section before selecting a response.

Materials & experimental systems

n/a	Involved in the study	
☒	☐	Antibodies
☒	☐	Eukaryotic cell lines
☒	☐	Palaeontology and archaeology
☒	☐	Animals and other organisms
☐	☒	Human research participants
☒	☐	Clinical data
☒	☐	Dual use research of concern

Methods

n/a	Involved in the study	
☒	☐	ChIP-seq
☒	☐	Flow cytometry
☒	☐	MRI-based neuroimaging

Human research participants

Policy information about studies involving human research participants

Population characteristics	This study included a total of 21 subjects with drug-resistant epilepsy (14 males; 25.0 ± 11.2 years old, mean ± standard deviation [SD] on the day of the experiment) from three sites. One subject participated twice because of a second surgery after an interval of 2 years (E07 and E11). The subjects were implanted with intracranial electrodes prior to the study for the purpose of treating their epilepsy (number of subdural electrodes: 64.4 ± 17.0; number of depth electrodes: 10.8 ± 10.0).
Recruitment	All participants were recruited from patients implanted with electrodes at three university sites (Osaka University, Nara Medical University and Juntendo University). Participants were recruited based on recommendations from the surgeons who placed the electrodes.
Ethics oversight	The IRB of each site examined and approved the study. The approved numbers are following. Osaka University Medical Hospital: Approval No. 14353, UMIN000017900; Juntendo University Hospital: Approval No. 18-164; Nara Medical University Hospital: Approval No. 2098.

Note that full information on the approval of the study protocol must also be provided in the manuscript.
